# Elucidation of inhibitory effects on metastatic sentinel lymph nodes of breast cancer during One-Step Nucleic Acid Amplification

**DOI:** 10.1038/s41598-018-25911-w

**Published:** 2018-05-15

**Authors:** Yoshiya Horimoto, Masahiko Tanabe, Saiko Kazuno, Yoshiki Miura, Kaoru Mogushi, Hiroshi Sonoue, Atsushi Arakawa, Kazunori Kajino, Toshiyuki Kobayashi, Mitsue Saito

**Affiliations:** 1 Department of Breast Oncology, Juntendo University School of Medicine, 3-1-3 Hongo, Bunkyo-ku, Tokyo 113-8431 Japan; 2 Department of Pathology and Oncology, Juntendo University School of Medicine, 3-1-3 Hongo, Bunkyo-ku, Tokyo 113-8431 Japan; 3 Department of Breast and Endocrine Surgery, Graduate School of Medicine, The University of Tokyo, 7-3-1 Hongo, Bunkyo-ku, Tokyo 113-8655 Japan; 4 Laboratory of Proteomics and Biomolecular Science, Research Support Center, Juntendo University Graduate School of Medicine, 2-1-1 Hongo, Bunkyo-ku, Tokyo 113-0033 Japan; 5 Diagnostics and Therapeutics of Intractable Diseases, Juntendo University Graduate School of Medicine, 2-1-1 Hongo, Bunkyo-ku, Tokyo 113-0033 Japan; 6 Department of Human Pathology, Juntendo University School of Medicine, 3-1-3 Hongo, Bunkyo-ku, Tokyo 113-8431 Japan

## Abstract

One-step nucleic acid amplification (OSNA) is an established method for intraoperative diagnosis of breast cancer metastasis in sentinel lymph nodes, based on quantification of *CK19* mRNA, specific to breast epithelial cells. Inhibitors interfere with the PCR amplification process of PCR. Thus, OSNA, based on gene amplification without RNA purification, might be impacted by numerous factors persisting in a sample, and thereby potentially acting as PCR inhibitors. However, neither the characteristics of breast cancers showing inhibitory effects during OSNA, nor any of the possible inhibitors, have as yet been identified. Inhibitory effects detected during OSNA in 72 metastatic lymph nodes and the patients’ clinicopathological features were examined. Left-over OSNA samples were analyzed with mass spectrometry to identify proteins possibly acting as inhibitors. Most tumors showed inhibitory effects, though to varying degrees. Large tumor, young age and high tumor-infiltrating lymphocyte counts were related to stronger inhibitory effects. Proteome analysis revealed elevations in RPB9 protein and EIF2 signaling upregulation in samples showing strong inhibitory effects. Tumors showing strong inhibitory effects had clinically relevant characteristics, including large size and extensive tumor-infiltrating lymphocyte involvement. Identifying inhibitors in OSNA might provide new insights into breast cancer biology as well as advancing the current technology.

## Introduction

### Role of OSNA method in breast cancer treatment

The one-step nucleic acid amplification (OSNA®) assay (Sysmex Corporation, Japan) is an established method for intraoperative diagnosis of breast cancer metastasis in axillary sentinel lymph nodes (SLNs)^1^. This assay is performed based on quantification of cytokeratin 19 (*CK19*) mRNA and has been shown to be more diagnostically accurate than classical pathological diagnosis using frozen sections^2,3^. Prediction of metastasis in non-sentinel lymph nodes is also potentially feasible with this method^4–7^.

OSNA has been widely introduced throughout Japan and many other countries, mostly in Europe, and is now being applied to clinical practice. It is also applicable to colon and gastric cancers^8,9^. Even with the recent preference for less radical surgical invasion for managing breast cancer, OSNA still has a role, e.g. in selecting patients in whom axillary dissection can be omitted, by providing more accurate evaluation of lymph node metastasis^10^.

### Principles of OSNA

OSNA detects *CK19* mRNA in sentinel lymph nodes. A node will be judged as harboring breast cancer metastasis when amplification of *CK19* mRNA is detected, because CK19 expression is specific to breast epithelial cells and theoretically does not exist in normal lymph nodes. RTqPCR generally requires several hours with multiple steps, including mRNA extraction, purification and cDNA synthesis, making intraoperative evaluation time-consuming. To reduce the time required for examination, OSNA employs the LAMP (Loop-mediated Isothermal Amplification) method. LAMP allows gene amplification without RNA purification and can be completed in a single step by incubating samples at a constant temperature with a mixture of primers and DNA polymerase^11^. Thereby, gene detection is completed within 30 to 40 minutes.

### Effects of PCR inhibitors on OSNA

It is widely known that the amplification process of PCR can be inhibited or prevented by several substances known as “inhibitors”^12^ and that inhibition is one of the most common causes of PCR failure^13^. The mechanism underlying this phenomenon is poorly understood, though multiple steps during the PCR process have been suggested to be involved (e.g. interference in DNA extraction, nucleic acid degradation, inhibition of polymerase activity)^12^. Generally, researchers overcome inhibitory effects in their experiments by devising specific protocols, e.g. employing several reagents to remove contaminants in order to obtain samples that are as pure as possible. Dilution of the inhibitors below their effective concentration is also a common approach^14^ and, indeed, PCR is restored when samples are diluted. However, it should be noted that even in highly purified samples, the effects of inhibitors might persist without significantly altering the PCR reaction.

### Definition of Plus I and its rank among the cases showing inhibitory effects

With the LAMP method, samples can be analyzed without RNA purification and results are obtained very quickly. However, numerous factors, i.e. impurities including proteins and DNA remaining in a sample, might act as PCR inhibitors. Previous reports have suggested inhibitory factors, such as hemoglobin^15^ and bile salts, to possibly exist in clinical samples^16,17^. However, to date, no inhibitors impacting OSNA analysis have been identified.

Inhibition of PCR by inhibitors in OSNA is usually minor because amplification of *CK19* is generally adequate in most cases. However, if a lymph node has only a small cancer nest, such as micrometastasis, inhibition might directly affect the diagnosis. OSNA categorizes positive results into 2 groups according to copy numbers of *CK19* (++, ≥5000 copies and +, ≥250 copies). Details of the diagnostic strategy provided by the manufacturer are shown in Table 1 and Supplementary Figure 1. Considering the possible existence of PCR inhibitors, diluted samples (1:10) are always tested simultaneously. If the *CK19* copy number is smaller than 5000 and the reaction is inhibited, the result will be displayed as “plus I” (Table 1 and Supplementary Figure 2).

**Table 1:** Diagnostic strategy for assessing OSNA results.

Copy number		CK19-D		
		<250	250≤ and <5000	≥5000
CK19	<250	No metastasis	**Plus I**	
	250≤ and <5000	Micro metastasis		**Plus I**
	≥5000	Macro metastasis		

The plus I rates reportedly range from 3 to 15%^3,5,6,18^. However, because only a few reports have provided plus I rates, the exact rate remains unknown. Plus I does not indicate the existence of contaminants because there is no inhibitory reaction in positive controls, while the existence of cancer cells is definite because *CK19* amplification has been confirmed in diluted samples. Plus I is clinically considered to represent “macrometastasis” because absolute values of the *CK19* copy number are no longer reliable, although there might be some cases with micrometastases among those who have Plus I results. To our knowledge, there have been no studies examining Plus I itself.

We speculate that PCR inhibitors would exist in any case with macrometastases, not only in those with Plus I results. Therefore, we examined all patients with lymph node (LN) metastasis diagnosed by OSNA to elucidate the characteristics of breast cancers that exert inhibitory effects on PCR during OSNA analysis. We also endeavored to identify the causes of such inhibitory effects.

## Results

### Inhibitory effects were observed in most cases

**Figure 1:**
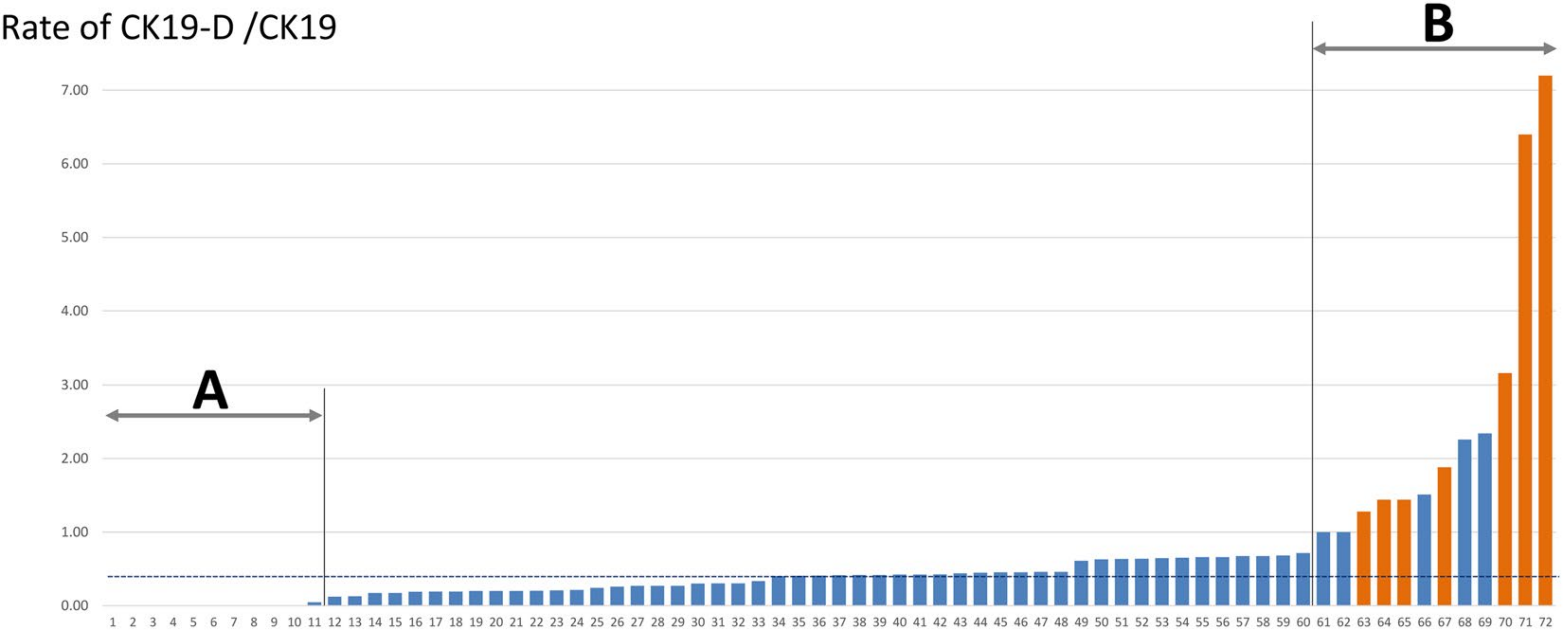
Inhibitory effects in the 72 patients. Orange bar indicates plus I cases, while dotted horizontal line indicates the median CK19-D/CK19 ratio, 0.41. Group A includes eleven cases showing no inhibitory effect, with a CK19-D/CK19 value of less than 0.1. Group B is comprised of 12 cases showing a strong inhibitory effect, as indicated by a CK19-D/CK19 value of 1.0 or more.

Most samples showed an inhibitory effect during OSNA, though to varying degrees (Fig. 1). The median rate of CK19-D/CK19 was 0.41. Eleven cases showed no inhibitory effect (Group A, indicated in Fig. 1), with CK19-D/CK19 values of less than 0.1. On the other hand, there were 12 cases showing strong inhibitory effects, as reflected by a CK19-D/CK19 value of 1.0 or higher (Group B).

### Tumors showing strong inhibitory effects had more malignant characteristics

Next, we divided all patients into two groups based on a cut-off value of 0.41, the median CK19-D/CK19 ratio (Fig. 1). Comparisons of clinicopathological features between the groups are shown in Supplementary Table 2. Furthermore, to elucidate the characteristics of tumors that showed strong inhibitory effects, we compared patients at both ends of the spectrum (indicated as Groups A and B with arrows in Fig. 1), the 11 patients with CK19-D/CK19 values of less than 0.1 and the 12 patients with values of 1.0 or higher (Table 2). When these typical subpopulations were compared, several trends emerged. Tumors showing strong inhibitory effects were more common in young patients (p = 0.033), were large (p = 0.028) and had more lymph node involvement (p = 0.034), as compared to those that showed no inhibitory effect. There were also tendencies for high grade, high Ki67 labelling index, and more tumor infiltrating lymphocyte (TIL) involvement to frequently be observed in tumors with CK19-D/CK19 values of 1.0 or higher, but the differences were not statistically significant. There were no differences among intrinsic subtypes. Next, we employed a multivariate logistic regression model to identify factors predicting strong inhibitory effects in 23 patients in total, from both Group A and Group B. Large tumor (p = 0.004), young age (p = 0.014) and extensive TIL (p = 0.024) were associated with strong inhibitory effects (Supplementary Table 3).

**Table 2:** Comparison of clinicopathological features between Groups A and B.

		CK19-D/CK19				*p value*
		Group A		Group B		
		<0.1		>1.0		
n		11		12		
Age	(range)	59	(40–76)	50	(39–77)	***0***.***033***
Histology	IDC (NST)	11	(100%)	10	(83%)	0.478
	Others*	0	(0%)	2	(17%)	
Tumor size (mm)	(range)	17.1	(0–37)	31.7	(0–75)	***0***.***028***
Number of metastatic LNs**	(range)	1.0	(1)	1.6	(1–4)	***0***.***034***
Ly (+)		0	(0%)	2	(17%)	0.478
High NG		1	(9%)	6	(50%)	0.069
Ki67 L.I. (%)	(range)	31.0	(5–60)	44.8	(12–80)	0.079
High TIL		1	(9%)	6	(50%)	0.069
ER (+)		10	(91%)	11	(92%)	1.000
PgR (+)		10	(91%)	11	(92%)	1.000
HER2 (3+)		3	(27%)	2	(17%)	0.640

*Other than NST. **Mean number of total metastatic LNs.

### Tumors showing strong inhibitory effects had variable protein expressions and pathway regulations

**Figure 2:**
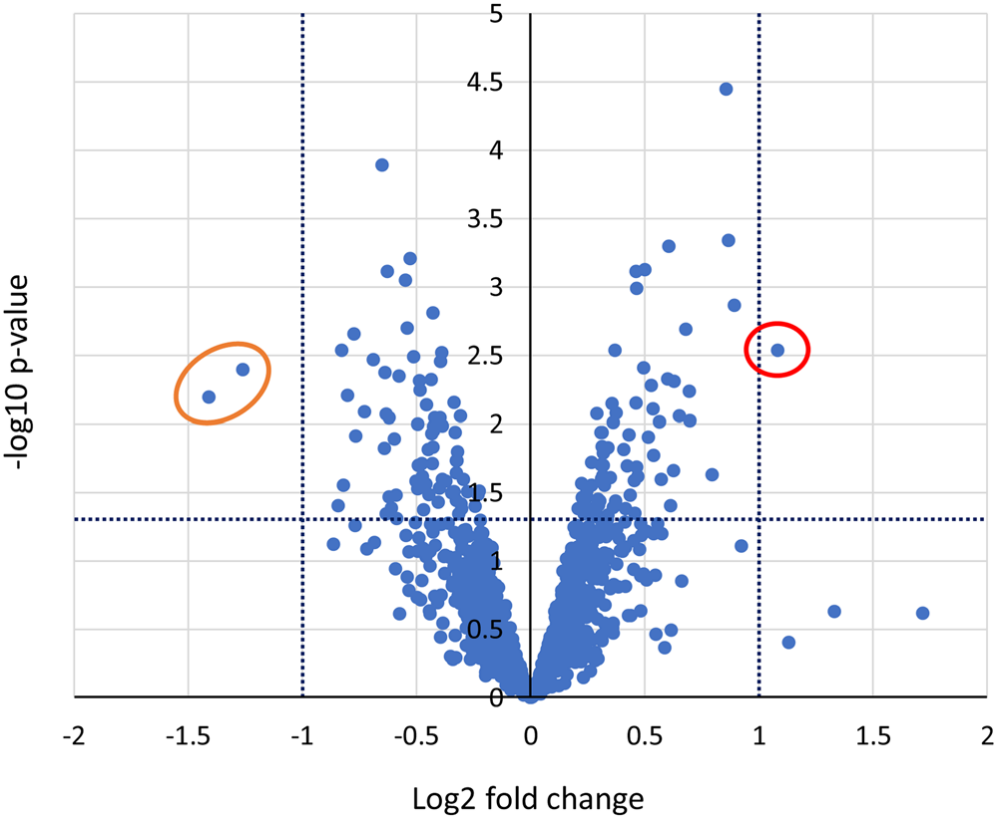
Volcano plots of proteins differed between the two groups. All 1228 proteins tested are plotted. The red circle indicates RPB9 protein, the only protein in Group B present at a statistically significantly higher amount. The orange circle indicates perforin-1 and the alpha-crystallin B chain, the expressions of which were reduced.

As noted above, left-over OSNA samples from 8 patients, 4 cases each from Groups A and B, were analyzed with mass spectrometry. Among 1228 proteins examined, LIMMA analysis revealed 83 proteins which differed significantly in their expression levels between the two groups. Thirty-eight proteins were highly expressed and 45 showed lower expressions in Group B than in Group A (shown in Supplementary Table 4). The top ten differentially expressed proteins among these are presented in Table 3. The volcano plot also revealed RPB9 to be the only protein, among all 1228 proteins tested, to be expressed at a statistically higher amount, while the expressions of perforin-1 and the alpha-crystallin B chain were lower (Fig. 2).

**Table 3:** Differentially expressed proteins in Group 2.

Protein	Name	Fold	p-value
RPB9_HUMAN	DNA-directed RNA polymerase II subunit RPB9	2.117	0.00290
RAC1_HUMAN	Ras-related C3 botulinum toxin substrate 1	1.854	0.00136
PCLI1_HUMAN	PTB-containing, cubilin and LRP1-interacting protein	1.822	0.00046
LGUL_HUMAN	Lactoylglutathione lyase	1.809	0.00004
RL32_HUMAN	60S ribosomal protein L32	1.620	0.00941
RS5_HUMAN	40S ribosomal protein S5	1.619	0.00579
PSMD8_HUMAN	26S proteasome non-ATPase regulatory subunit 8	1.601	0.00204
SYPL1_HUMAN	Synaptophysin-like protein 1	1.569	0.00873
PP2AA_HUMAN	Serine/threonine-protein phosphatase 2A catalytic subunit alpha isoform	1.545	0.00488
PML_HUMAN	Protein PML	1.519	0.00050
**Protein**	**Name**	**Fold**	**p-value**
CRYAB_HUMAN	Alpha-crystallin B chain	−2.657	0.00635
PERF_HUMAN	REVERSED Perforin-1	−2.398	0.00403
EMAL4_HUMAN	Echinoderm microtubule-associated protein-like 4	−1.777	0.00289
F213A_HUMAN	Redox-regulatory protein FAM213A	−1.746	0.00620
ULA1_HUMAN	NEDD8-activating enzyme E1 regulatory subunit	−1.710	0.00220
WIPI2_HUMAN	WD repeat domain phosphoinositide-interacting protein 2	−1.701	0.01217
SERA_HUMAN	D-3-phosphoglycerate dehydrogenase	−1.656	0.00818
TRY1_HUMAN	Trypsin-1	−1.613	0.00341
PSB3_HUMAN	Proteasome subunit beta type-3	−1.571	0.00013
ARHG2_HUMAN	Rho guanine nucleotide exchange factor 2	−1.560	0.01502

**Figure 3:**
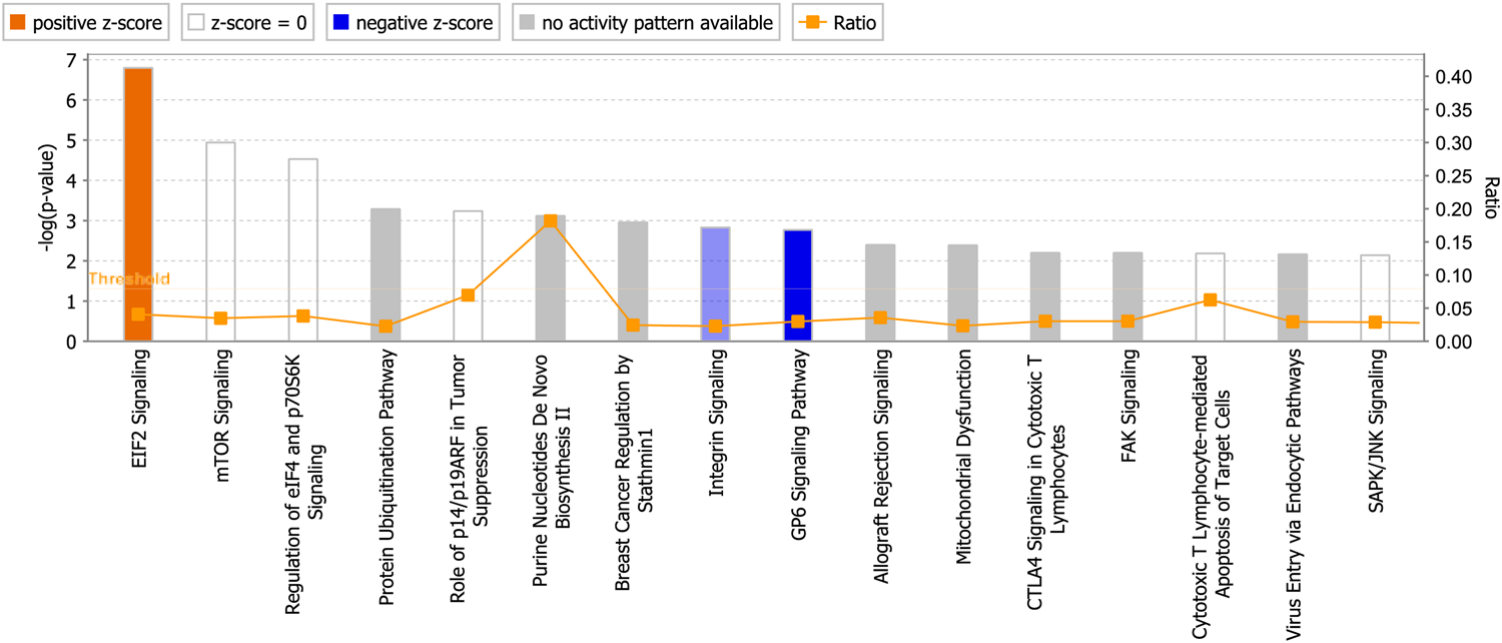
Canonical pathways varied in Group 2. Orange indicates an “activated” canonical pathway, while blue means “inactivated”. White: activation not predictable in any particular direction, grey: not covered for predictive function, Ratio: ratio of the molecules examined to those that are already known.

Next, we conducted pathway analysis using the 83 proteins. Canonical pathways are shown in Fig. 3. EIF2 (eukaryotic initiation factor 2) signaling was upregulated in Group 2. We also observed the integrin signaling and the GP6 (glycoprotein VI) signaling pathways to be downregulated. As indicated in Fig. 3, major signaling pathways such as mTOR and JNK, and also those related to immune responses such as “Cytotoxic T lymphocyte-mediated apoptosis” were detected as having been activated but not in any particular direction.

## Discussion

To our knowledge, this study is the first to investigate the relationships between inhibitory reactions in OSNA samples and clinicopathological features. We confirmed that the inhibitory effect is observed in most tumors, though to varying degrees.

Strong inhibitory effects were more common in younger patients. We speculate that differences in serum estradiol levels according to age might be one of the factors impacting these effects, though the mechanism is unknown. We further investigated premenopausal patients in Groups A and B (3 and 9 patients, respectively) to determine whether they were in the luteal phase on the day of surgery, since we previously observed a Ki67 labelling index increase in patients who were menstruating^19^. Interestingly, the proportion of patients in the luteal phase in Group B was higher than that in Group A (66% and 33%, respectively), although the number of patients examined was too small to allow meaningful conclusions to be drawn.

Also, large tumor burden appears to be associated with an inhibitory effect, as Group B had larger tumors and more lymph node metastases. Tumor size was an independent factor predicting a strong inhibitory effect (p = 0.004). Large tumors may have more heterogeneous tumor cells, as compared to relatively monotonous breast cancers^20,21^, and would thus unusually express a variety of proteins. We might have detected novel inhibitory factors among the large variety of proteins examined in this study.

As to the malignant propensity of the tumor itself, tumors in Group B were higher grade and had higher Ki67 labelling indices, although the differences were not statistically significant. Extensive TIL was also an independent factor predicting a strong inhibitory effect (p = 0.024). We speculate that a tumor with high malignancy might have more turnover of cancer cells and would thereby trigger immune responses against metastatic disease. EIF2 signaling particularly involves the first step of protein synthesis in mRNA translation. Upregulation of this signaling in Group B might simply explain this high cell turnover, not only of cancer cells but also of a variety of immune cells present in lymph nodes.

Highly expressed proteins in Group B were related to transcriptional regulation, cell cycle regulation, and metabolism. Substances exerting an inhibitory effect are not necessarily proteins. Furthermore, several substances may exist in an OSNA sample. However, it is very interesting that RPB9, the most highly expressed protein in Group B, is a component of RNA polymerase. It might interfere with DNA polymerase in the process of synthesizing *CK19* cDNA during OSNA. Target-specific analyses should be conducted in the future to identify materials causing inhibitory effects. Experiments to test whether removal of RBP9 protein diminish inhibitory effects are eagerly awaited and we plan to use such an approach in future studies.

We could not determine whether the possible inhibitors identified herein might act generally in PCR or be specific to the LAMP method. We speculate that the latter is more likely, considering that there are several specific processes comprising the LAMP method, including the extension reaction using double stranded DNA as a template after cDNA has been synthesized and formed a loop structure.

Our data revealed that a tumor showing a strong inhibitory effect has clinically relevant characteristics, such as large size and extensive TIL involvement. Tumor heterogeneity might also be associated with inhibitory effects. Further investigation with more materials is needed in the future to identify inhibitors impacting OSNA, to conduct more precise quantification of LN metastasis and advance the current technology (e.g. new OSNA targeting mRNAs other than that of *CK19*). Also, identifying inhibitors might provide new insights, for future research, into breast cancer biology.

## Methods

### Clinical samples

There were 442 patients (821 LNs in total) who underwent surgery with OSNA at our institution during the January 2013 through December 2015 period. All patients were diagnosed clinically as N0 prior to surgery. Seventy-two patients (100 LNs in total) were eventually diagnosed as having LN metastasis by OSNA. We enrolled these 72 cases and investigated the inhibitory effects observed during OSNA in detail. Clinicopathological features of the 72 cases are shown in Supplementary Table 1. There were seven Plus I cases among the 72. This study was carried out with approval from the ethics committee of Juntendo University Hospital (no: 17-012) and all specimens were collected after obtaining informed consent from the patients. All experiments were performed in accordance with relevant guidelines and regulations.

### OSNA analysis and definition of inhibitory reaction

First, SLNs were detected employing standard methods after injection of 99mTc-phytate (radioactive colloid) and indigo carmine (blue dye) into the skin of the breast. OSNA was intraoperatively performed according to the manufacturer’s instructions and the results were assessed, as shown in Table 1 and Supplementary Figure 1. For this study, we retrospectively analyzed raw data for copy numbers of *CK19*. Diluted samples (1 in 10) were also simultaneously measured, indicated as *CK19-D*. Theoretically, the rate of CK19-D/CK19 should be 0.1. The value would exceed 0.1 if even very slight inhibition is exerted by inhibitory factors. In assessing Plus I cases, a cut-off value of 1.0 was automatically used as a default setting by the manufacturer. The CK19-D copy number in such cases is the inverse of that of CK19, meaning that there is an apparent inhibitory reaction during OSNA analysis. In this study, we defined a case with a CK19-D/CK19 value of 0.1 or less as showing “no” inhibitory effect. We also calculated the CK19-D/CK19 ratio for all samples. However, absolute CK19-D/CK19 values could not be calculated in Plus I cases because the CK19-D value is available only as “250 copies or less”, displayed as the result (Supplementary Figure 2). Thus, we employed 250 as the tentative CK19-D value in such cases, such that the minimum CK19-D/CK19 values were used for analysis. In patients with several metastatic SLNs, the SLN with the highest CK19 copy number was selected for application in the current study.

### Pathological examination

Pathological examinations for primary tumors were carried out at Juntendo University Hospital by two experienced pathologists. Nuclear grade (NG) was judged based on the modified Bloom-Richardson histological grades. Estrogen receptor (ER) status and progesterone receptor (PgR) status were assessed semi-quantitatively and reported as positive when more than 1% of the nuclei of cancer cells showed staining. HER2 was judged to be positive if more than 10% of tumor cells showed strong staining of the entire cell membrane, or HER2/neu gene amplification was confirmed by fluorescence *in situ* hybridization. As to Ki67, a hot spot was chosen under 200× magnification and cells positive for nuclear Ki67 were then assessed semi-quantitatively.

To reveal the characteristics of breast cancers exerting an inhibitory effect on PCR, we also assessed TILs in the primary lesion, speculating that stromal reactions including TILs might produce more numerous reactions including immune responses involving SLN. The TIL evaluation was performed under 200× magnification at the edge of the primary tumor and the percentage of stromal TIL was assessed, following the guidelines established by the International TIL Working Group 2014^22^. In the current study, “high” TIL was defined as a tumor with more than 30% stromal TIL.

### Proteomic analysis

Left-over OSNA samples from 8 patients, 4 samples each showing no and strong inhibitory effects, were analyzed with mass spectrometry. For comprehensive analysis, we employed the iTRAQ® (AB Sciex, Framingham, MA) system. After purification with dialysis, 100 μg of proteins in remnant OSNA samples from 8 patients in total were digested with trypsin and then labelled with an iTRAQ reagents-8plex kit according to the manufacturer’s protocol. The digested peptides of 4 samples showing no inhibitory effect in OSNA analysis were labelled with 113, 114, 115, and 116 iTRAQ reagents, respectively, while the other 4 samples that showed a strong inhibitory effect were labelled with 117, 118, 119, and 121. Samples were then fractionated into 8 fractions using a Pierce High pH Reversed-phase peptide fractionation kit (Thermo Scientific, IL). Each fraction was dried with a vacuum centrifuge concentrator and dissolved in 0.1% TFA. Mass spectrometric analysis was performed with the Triple TOF 5600 operated with Analyst TF 1.7 software (AB Sciex). The Triple TOF 5600 was connected to a Eksigent nano LC system with attached nano cHiPLC trap column chrom XP C18-CL (200 μm × 0.5 mm) and nano cHiPLC column chrom XP C18-CL (75 μm × 15 cm). The solvent system consisted of solution A: 0.1% (v/v) formic acid, and solution B: 80% (v/v) acetonitrile containing 0.1% (v/v) formic acid. The column was eluted with a linear gradient from 0% (v/v) to 40% (v/v) of B in 100 min. The flowrate was 300 nl/min. Mass spectrometric data were analyzed with Protein Pilot 5.0 software (AB Sciex) using the Uniprot database (release 8/30/2016). A confidence cut-off value of 1% FDR (false discovery rate) was applied for protein identification. Data were then analyzed employing QIAGEN’s Ingenuity® Pathway Analysis (IPA®, QIAGEN Redwood City, www.qiagen.com/ingenuity).

### Statistical analysis

Statistical analyses were performed using JMP 11.2.1 statistical software (SAS Institute Inc., Cary, NC, USA). Associations between clinicopathological parameters and inhibitory effects were evaluated using Fisher’s exact test. For comparisons of mean values, such as those for age and tumor size, the two-sided Welch’s t-test was carried out. A p-value < 0.05 was considered to indicate a statistically significant difference. We applied a logistic regression model to our data and used the backward procedure with the minimum Akaike information criterion (AIC). We conducted a preliminary principal component analysis that allows grouping of all variables. Based on the results obtained, age, tumor size, NG, and TIL were selected as full-model variables for the present analysis. For comparison of protein expressions obtained from mass spectrometry, LIMMA analysis was performed employing R software^23^. In the LIMMA analysis, data were adjusted considering the FDR with 0.30 as the cut-off value. Thus, only a difference with a p-value < 0.05, FDR < 0.30, and an absolute fold-change >1.5 was judged to be statistically significant.

### Data Availability

No datasets were generated or analyzed during the current study.

## Electronic supplementary material

Supplementary figures and tables
