# Supplementary material for: Elucidation of inhibitory effects on metastatic sentinel lymph nodes of breast cancer during One-Step Nucleic Acid Amplification
Source: Sci Rep. 2018 May 15;8:7563. doi: 10.1038/s41598-018-25911-w (PMC5954024; doi:10.1038/s41598-018-25911-w)
Supplement: Supplementary file 1 — Supplementary figures and tables [file 41598_2018_25911_MOESM1_ESM.pdf]

Title: **Elucidation of inhibitory effects on metastatic sentinel lymph nodes of breast cancer during One-Step Nucleic Acid Amplification**

Author names:
Yoshiya Horimoto, Masahiko Tanabe, Saiko Kazuno, Yoshiki Miura, Kaoru Mogushi, Hiroshi Sonoue, Atsushi Arakawa, Kazunori Kajino, Toshiyuki Kobayashi, Mitsue Saito

Supplementary Figure 1. Representative OSNA results of metastatic lymph nodes

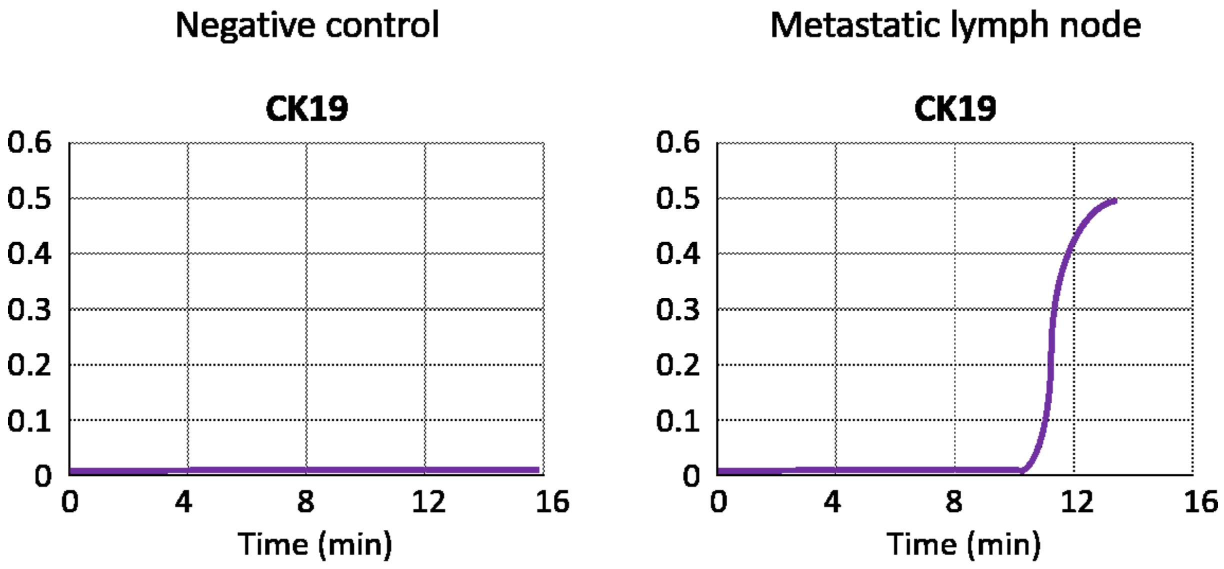

Time	ND [min]
Conc.	<2.5e+02 [copies/uL]
Qualitative	(-)

Time	10.6 [min]
Conc.	**2.5e+03** [copies/uL]
Qualitative	(+)

In a metastatic lymph node, amplification of *CK19* mRNA is evidenced by CK19 copy numbers of 250 or more. This case shows 2500 copies, meaning micro-metastasis (see Table 1 as well).

Supplementary Figure 2. Explanation of Plus I results

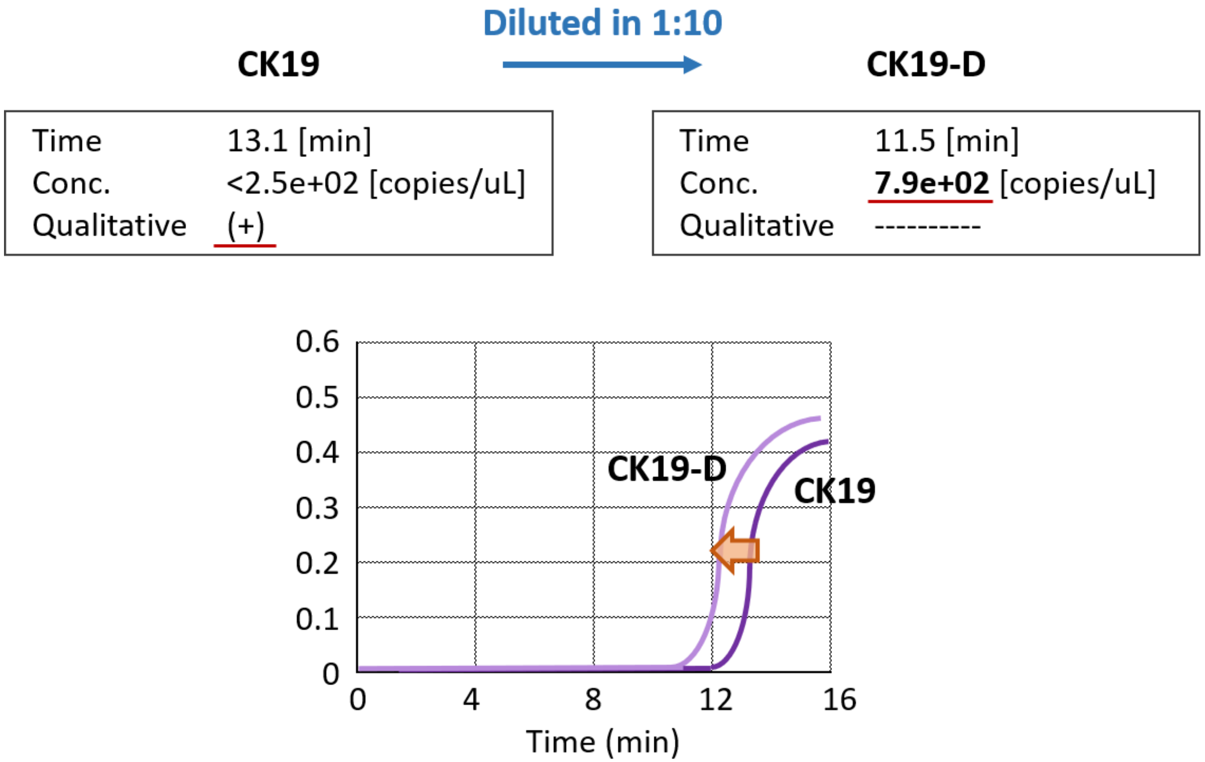

In a Plus I case, the CK19-D copy number exceeds CK-19, indicating an improvement of mRNA amplification as a result of sample dilution. The original CK-19 copy number is less than 250 due to inhibitory effects. The leftward shift of the CK-19 curve reflects this phenomenon.

Supplementary Table 1. Clinicopathological features of the 72 cases

n		72	
Age	(range)	52	(38-81)
Histology	IDC (NST)	61	(85%)
	Others*†	11	(15%)
Tumor size (mm)	(range)†	22	(0-75)
Number of metastatic LNs**	(range)	2.2	(1-7)
Ly (+)		19	(26%)
High NG		12	(17%)
Ki67 L.I. (%)	(range)	30	(5-90)
ER (+)		66	(92%)
PgR (+)		63	(88%)
HER2 (3+)		10	(14%)

*invasive cancer other than NST, such as mucinous carcinoma.

†One patient had only DCIS.

**Mean values (median: 2.0 (1-11))

NST: no special type

Supplementary Table 2. Clinicopathological features according to inhibitory effects

		CK19-D/CK19				
		≤0.41		>0.41		*p value*
n		37		35		
Age	(range)	55	(39-81)	49	(38-82)	n.s.
Histology	IDC (NST)	32	(86%)	30	(86%)	n.s.
	Others*	5	(14%)	5	(14%)	
Tumor size (mm)	(range)	25.7	(0-73)	24.8	(1-70)	n.s.
Number of metastatic LNs**	(range)	1.7	(1-7)	1.4	(1-4)	n.s.
Ly (+)		9	(24%)	10	(29%)	n.s.
High NG		5	(14%)	7	(20%)	n.s.
Ki67 L.I. (%)	(range)	35.6	(5-90)	37.7	(5-90)	n.s.
High TIL		13	(35%)	16	(46%)	n.s.
ER (+)		34	(92%)	32	(91%)	n.s.
PgR (+)		33	(89%)	30	(86%)	n.s.
HER2 (3+)		4	(11%)	6	(17%)	n.s.

*other than NST

**Mean number of total metastatic LNs

Supplementary Table 3. Logistic model for predicting strong inhibitory effect

Variables	OR	95% CI	P value
Tumor size	1.2×10^{7}*	$20.56 - 3.95 \times 10^{18}$*	**0.004**
Age	4.3×10^{3}*	$3.44 - 3.84 \times 10^{9}$*	**0.014**
High TIL	49.2	$1.51 - 6.43 \times 10^{4}$	**0.024**
High NG	33.4	$0.98 - 8.99 \times 10^{3}$	0.052

OR: odds ratio, CI: confidence interval, *Range of odds ratios

Protein	Name	Fold	p-value
RPB9_HUMAN	DNA-directed RNA polymerase II subunit RPB9 OS=Homo sapiens GN=POLR2I PE=1 SV=1	2.117	0.00290
RAC1_HUMAN	Ras-related C3 botulinum toxin substrate 1 OS=Homo sapiens GN=RAC1 PE=1 SV=1	1.854	0.00136
PCLI1_HUMAN	PTB-containing, cubilin and LRP1-interacting protein OS=Homo sapiens GN=PID1 PE=1 SV=1	1.822	0.00046
LGUL_HUMAN	Lactoylglutathione lyase OS=Homo sapiens GN=GLO1 PE=1 SV=4	1.809	0.00004
RL32_HUMAN	60S ribosomal protein L32 OS=Homo sapiens GN=RPL32 PE=1 SV=2	1.620	0.00941
RS5_HUMAN	40S ribosomal protein S5 OS=Homo sapiens GN=RPS5 PE=1 SV=4	1.619	0.00579
PSMD8_HUMAN	26S proteasome non-ATPase regulatory subunit 8 OS=Homo sapiens GN=PSMD8 PE=1 SV=2	1.601	0.00204
SYPL1_HUMAN	Synaptophysin-like protein 1 OS=Homo sapiens GN=SYPL1 PE=1 SV=1	1.569	0.00873
PP2AA_HUMAN	Serine/threonine-protein phosphatase 2A catalytic subunit alpha isoform OS=Homo sapiens GN=PPP2CA PE=1 SV=1	1.545	0.00488
PML_HUMAN	Protein PML OS=Homo sapiens GN=PML PE=1 SV=3	1.519	0.00050
COX41_HUMAN	Cytochrome c oxidase subunit 4 isoform 1, mitochondrial OS=Homo sapiens GN=COX4I1 PE=1 SV=1	1.516	0.00468
PSA5_HUMAN	Proteasome subunit alpha type-5 OS=Homo sapiens GN=PSMA5 PE=1 SV=3	1.478	0.00964
FUBP3_HUMAN	Far upstream element-binding protein 3 OS=Homo sapiens GN=FUBP3 PE=1 SV=2	1.451	0.01686
DDX5_HUMAN	Probable ATP-dependent RNA helicase DDX5 OS=Homo sapiens GN=DDX5 PE=1 SV=1	1.451	0.00778
STX7_HUMAN	Isoform 2 of Syntaxin-7 OS=Homo sapiens GN=STX7	1.443	0.00525
CPPED_HUMAN	Serine/threonine-protein phosphatase CPPED1 OS=Homo sapiens GN=CPPED1 PE=1 SV=3	1.430	0.01250
NIT2_HUMAN	Omega-amidase NIT2 OS=Homo sapiens GN=NIT2 PE=1 SV=1	1.415	0.00075
GNPI2_HUMAN	Glucosamine-6-phosphate isomerase 2 OS=Homo sapiens GN=GNPDA2 PE=1 SV=1	1.408	0.00391
YBOX2_HUMAN	Y-box-binding protein 2 OS=Homo sapiens GN=YBX2 PE=1 SV=2	1.379	0.00102
IF4A2_HUMAN	Eukaryotic initiation factor 4A-II OS=Homo sapiens GN=EIF4A2 PE=1 SV=2	1.376	0.00707
QCR1_HUMAN	Cytochrome b-c1 complex subunit 1, mitochondrial OS=Homo sapiens GN=UQCRC1 PE=1 SV=3	1.376	0.00076
PSA2_HUMAN	Proteasome subunit alpha type-2 OS=Homo sapiens GN=PSMA2 PE=1 SV=2	1.347	0.01193
DEFI6_HUMAN	Differentially expressed in FDCP 6 homolog OS=Homo sapiens GN=DEF6 PE=1 SV=1	1.340	0.02008
SNX5_HUMAN	Sorting nexin-5 OS=Homo sapiens GN=SNX5 PE=1 SV=1	1.325	0.01527
COMD5_HUMAN	COMM domain-containing protein 5 OS=Homo sapiens GN=COMMD5 PE=1 SV=1	1.296	0.00830
GSTP1_HUMAN	Glutathione S-transferase P OS=Homo sapiens GN=GSTP1 PE=1 SV=2	1.291	0.00291
NLTP_HUMAN	Non-specific lipid-transfer protein OS=Homo sapiens GN=SCP2 PE=1 SV=2	1.284	0.00974
RL30_HUMAN	60S ribosomal protein L30 OS=Homo sapiens GN=RPL30 PE=1 SV=2	1.280	0.00711
PROF1_HUMAN	Profilin-1 OS=Homo sapiens GN=PFN1 PE=1 SV=2	1.263	0.01485
RS15A_HUMAN	40S ribosomal protein S15a OS=Homo sapiens GN=RPS15A PE=1 SV=2	1.251	0.01555
RL10A_HUMAN	60S ribosomal protein L10a OS=Homo sapiens GN=RPL10A PE=1 SV=2	1.247	0.02005
TBB5_HUMAN	Tubulin beta chain OS=Homo sapiens GN=TUBB PE=1 SV=2	1.246	0.01618
NPM_HUMAN	Nucleophosmin OS=Homo sapiens GN=NPM1 PE=1 SV=2	1.242	0.01151
LA_HUMAN	Lupus La protein OS=Homo sapiens GN=SSB PE=1 SV=2	1.241	0.01451
CLPP_HUMAN	ATP-dependent Clp protease proteolytic subunit, mitochondrial OS=Homo sapiens GN=CLPP PE=1 SV=1	1.237	0.01983
PGAM1_HUMAN	Phosphoglycerate mutase 1 OS=Homo sapiens GN=PGAM1 PE=1 SV=2	1.237	0.01152
PARK7_HUMAN	Protein DJ-1 OS=Homo sapiens GN=PARK7 PE=1 SV=2	1.223	0.00836
CATC_HUMAN	Dipeptidyl peptidase 1 OS=Homo sapiens GN=CTSC PE=1 SV=2	1.202	0.01903

Protein	Name	Fold	p-value
CRYAB_HUMAN	Alpha-crystallin B chain OS=Homo sapiens GN=CRYAB PE=1 SV=2	-2.657	0.00635
PERF_HUMAN	REVERSED Perforin-1 OS=Homo sapiens GN=PRF1 PE=1 SV=1	-2.398	0.00403
EMAL4_HUMAN	Echinoderm microtubule-associated protein-like 4 OS=Homo sapiens GN=EML4 PE=1 SV=3	-1.777	0.00289
F213A_HUMAN	Redox-regulatory protein FAM213A OS=Homo sapiens GN=FAM213A PE=1 SV=3	-1.746	0.00620
ULA1_HUMAN	NEDD8-activating enzyme E1 regulatory subunit OS=Homo sapiens GN=NAE1 PE=1 SV=1	-1.710	0.00220
WIPI2_HUMAN	WD repeat domain phosphoinositide-interacting protein 2 OS=Homo sapiens GN=WIPI2 PE=1 SV=1	-1.701	0.01217
SERA_HUMAN	D-3-phosphoglycerate dehydrogenase OS=Homo sapiens GN=PHGDH PE=1 SV=4	-1.656	0.00818
TRY1_HUMAN	Trypsin-1 OS=Homo sapiens GN=PRSS1 PE=1 SV=1	-1.613	0.00341
PSB3_HUMAN	Proteasome subunit beta type-3 OS=Homo sapiens GN=PSMB3 PE=1 SV=2	-1.571	0.00013
ARHG2_HUMAN	Rho guanine nucleotide exchange factor 2 OS=Homo sapiens GN=ARHGEF2 PE=1 SV=4	-1.560	0.01502
SHLB1_HUMAN	Endophilin-B1 OS=Homo sapiens GN=SH3GLB1 PE=1 SV=1	-1.557	0.00422
GRHPR_HUMAN	Isoform 2 of Glyoxylate reductase/hydroxypyruvate reductase OS=Homo sapiens GN=GRHPR	-1.551	0.00847
RL35_HUMAN	60S ribosomal protein L35 OS=Homo sapiens GN=RPL35 PE=1 SV=2	-1.545	0.00077
PRELP_HUMAN	Prolargin OS=Homo sapiens GN=PRELP PE=1 SV=1	-1.538	0.00898
TETN_HUMAN	Tetranectin OS=Homo sapiens GN=CLEC3B PE=1 SV=3	-1.512	0.01288
MINK1_HUMAN	Misshapen-like kinase 1 OS=Homo sapiens GN=MINK1 PE=1 SV=2	-1.492	0.00447
RM12_HUMAN	39S ribosomal protein L12, mitochondrial OS=Homo sapiens GN=MRPL12 PE=1 SV=2	-1.463	0.00089
B2MG_HUMAN	Beta-2-microglobulin OS=Homo sapiens GN=B2M PE=1 SV=1	-1.455	0.00199
CAN1_HUMAN	Calpain-1 catalytic subunit OS=Homo sapiens GN=CAPN1 PE=1 SV=1	-1.442	0.00062
AOC3_HUMAN	Membrane primary amine oxidase OS=Homo sapiens GN=AOC3 PE=1 SV=3	-1.427	0.00323
LAMA4_HUMAN	Laminin subunit alpha-4 OS=Homo sapiens GN=LAMA4 PE=1 SV=4	-1.410	0.01007
AP3B1_HUMAN	AP-3 complex subunit beta-1 OS=Homo sapiens GN=AP3B1 PE=1 SV=3	-1.408	0.01993
AL1A1_HUMAN	Retinal dehydrogenase 1 OS=Homo sapiens GN=ALDH1A1 PE=1 SV=2	-1.403	0.00486
RASA3_HUMAN	Ras GTPase-activating protein 3 OS=Homo sapiens GN=RASA3 PE=1 SV=3	-1.401	0.00568
TPM2_HUMAN	Isoform 2 of Tropomyosin beta chain OS=Homo sapiens GN=TPM2	-1.393	0.01948
ITA7_HUMAN	Integrin alpha-7 OS=Homo sapiens GN=ITGA7 PE=1 SV=3	-1.372	0.00724
INF2_HUMAN	Inverted formin-2 OS=Homo sapiens GN=INF2 PE=1 SV=2	-1.365	0.01535
DHB11_HUMAN	Estradiol 17-beta-dehydrogenase 11 OS=Homo sapiens GN=HSD17B11 PE=1 SV=3	-1.353	0.00473
C1QB_HUMAN	Complement C1q subcomponent subunit B OS=Homo sapiens GN=C1QB PE=1 SV=3	-1.350	0.01175
ACOC_HUMAN	Cytoplasmic aconitate hydratase OS=Homo sapiens GN=ACO1 PE=1 SV=3	-1.348	0.01938
CO6A2_HUMAN	Collagen alpha-2(VI) chain OS=Homo sapiens GN=COL6A2 PE=1 SV=4	-1.346	0.00154
PLIN1_HUMAN	Perilipin-1 OS=Homo sapiens GN=PLIN1 PE=1 SV=2	-1.346	0.01472
ABHDA_HUMAN	Mycophenolic acid acyl-glucuronide esterase, mitochondrial OS=Homo sapiens GN=ABHD10 PE=1 SV=1	-1.344	0.01044
GLYG_HUMAN	Glycogenin-1 OS=Homo sapiens GN=GYG1 PE=1 SV=4	-1.344	0.01114
PUR2_HUMAN	Trifunctional purine biosynthetic protein adenosine-3 OS=Homo sapiens GN=GART PE=1 SV=1	-1.337	0.00903
IF4A1_HUMAN	Eukaryotic initiation factor 4A-I OS=Homo sapiens GN=EIF4A1 PE=1 SV=1	-1.318	0.00900
DOA_HUMAN	HLA class II histocompatibility antigen, DO alpha chain OS=Homo sapiens GN=HLA-DOA PE=1 SV=1	-1.315	0.00350
DGKA_HUMAN	Diacylglycerol kinase alpha OS=Homo sapiens GN=DGKA PE=1 SV=3	-1.310	0.00301
GRB2_HUMAN	Growth factor receptor-bound protein 2 OS=Homo sapiens GN=GRB2 PE=1 SV=1	-1.308	0.01035
AIMP1_HUMAN	Isoform 2 of Aminoacyl tRNA synthase complex-interacting multifunctional protein 1 OS=Homo sapiens GN=AIMP1	-1.263	0.00699
CNN1_HUMAN	Calponin-1 OS=Homo sapiens GN=CNN1 PE=1 SV=2	-1.258	0.01154
S10A4_HUMAN	Protein S100-A4 OS=Homo sapiens GN=S100A4 PE=1 SV=1	-1.254	0.01848
VAMP2_HUMAN	Vesicle-associated membrane protein 2 OS=Homo sapiens GN=VAMP2 PE=1 SV=3	-1.251	0.01845
TKT_HUMAN	Transketolase OS=Homo sapiens GN=TKT PE=1 SV=3	-1.249	0.01600
PURA2_HUMAN	Adenylosuccinate synthetase isozyme 2 OS=Homo sapiens GN=ADSS PE=1 SV=3	-1.239	0.00876

Supplementary Table 4. All differentially expressed proteins in Group 2
